# New Phylogenetic Groups of Torque Teno Virus Identified in Eastern Taiwan Indigenes

**DOI:** 10.1371/journal.pone.0149901

**Published:** 2016-02-22

**Authors:** Kuang-Liang Hsiao, Li-Yu Wang, Chiung-Ling Lin, Hsin-Fu Liu

**Affiliations:** 1 Department of Bioscience and Biotechnology, National Taiwan Ocean University, Keelung, Taiwan; 2 Department of Medical Research, Mackay Memorial Hospital, Taipei, Taiwan; 3 Department of Medicine, Mackay Medical College, New Taipei City, Taiwan; 4 Department of Nursing, National Taipei University of Nursing and Health Sciences, Taipei, Taiwan; The University of Hong Kong, HONG KONG

## Abstract

Torque teno virus (TTV) is a single-stranded DNA virus highly prevalent in the world. It has been detected in eastern Taiwan indigenes with a low prevalence of 11% by using N22 region of which known to underestimate TTV prevalence excessively. In order to clarify their realistic epidemiology, we re-analyzed TTV prevalence with UTR region. One hundred and forty serum samples from eastern Taiwanese indigenous population were collected and TTV DNA was detected in 133 (95%) samples. Direct sequencing revealed an extensive mix-infection of different TTV strains within the infected individual. Entire TTV open reading frame 1 was amplified and cloned from a TTV positive individual to distinguish mix-infected strains. Phylogenetic analysis showed eleven isolates were clustered into a monophyletic group that is distinct from all known groups. In addition, another our isolate was clustered with recently described Hebei-1 strain and formed an independent clade. Based on the distribution pattern of pairwise distances, both new clusters were placed at phylogenetic group level, designed as the 6th and 7th phylogenetic group. In present study, we showed a very high prevalence of TTV infection in eastern Taiwan indigenes and indentified new phylogenetic groups from the infected individual. Both intra- and inter-phylogenetic group mix-infections can be found from one healthy person. Our study has further broadened the field of human TTVs and proposed a robust criterion for classification of the major TTV phylogenetic groups.

## Introduction

During the search for possible pathogens of non-A to E hepatitis, a novel DNA virus was discovered and named TT virus after the initials of the index patient [1]. In 2005, the International Committee on Taxonomy of Viruses (ICTV) officially designated TTV as Torque teno virus (TTV) deriving from the Latin terms torque meaning “necklace” and tenuis meaning “thin”, and classified it into a novel floating genus, Anellovirus [2]. TTV is the first human virus with a circular negative-stranded DNA genome [3]. Its genome is around 3.8k nucleotides (nt) in length including a well conserved untranslated region (UTR) and highly diverse coding regions [3,4]. Putative ORF1 is the longest ORF of TTV which covering around two-thirds of entire viral genome. Based on the molecular phylogeny of ORF1, human TTVs are currently classified into 4 or 5 phylogenetic groups [5–7]. TTV is circulating ubiquitously in many geographic regions; however, the worldwide prevalence of TTV was very diverse depending on the target genomic region for PCR detection and the clinical consequence of TTV infection remains unknown [4,8,9].

TTV prevalence has been reported in Taiwan, including the eastern Taiwanese indigenes, by using N22 region [10]. However, it has been shown that this genomic region will underestimate TTV prevalence severely [8]. To clarify their realistic epidemiology, we re-analyzed TTV prevalence with UTR region from eastern Taiwanese indigenes. TTV ORF1, covering around 2/3 of complete genome, can provide sufficient sequence information to allow between group comparisons and to represent a consistent phylogenetic classification [11]. We therefore amplified and used entire ORF1 for phylogenetic analysis to investigate their phylogenetic relationship with other TTV strains from all over the world.

## Materials and Methods

### Serum sample and DNA extraction

The study protocol was approved by the Institutional Review Board of Tzu Chi Medical Center and signed consent was obtained from each participant. A random sample of 140 adults aged 40-to-69 years was recruited from Gungfu Township, Hualien County during Dec 2003 to Apr 2004. Blood samples are collected in a portable refrigerator from 140 eastern Taiwanese indigenous, delivered to the central laboratory for serum separation, and stored at -70°C till processed. Viral DNA was extracted from 100 μl of serum by High Pure Viral Nucleic Acid Kit (Roche).

### Detection of TTV DNA

TTV DNA was detected via PCR amplification of viral 5’-UTR by using sense primer 5’-GCA CTT CCG AAT GGC TGA GT-3’ and antisense primer 5’-GWG CCT TGC CCR BRG CCC-3’. The amplicon is according to nucleotide position 97–252 of prototype TA278. Reaction was performed with AmpliTaq Gold DNA Polymerase (Applied Biosystems) in GeneAmp PCR System 9700 (Applied Biosystems) and using the following thermocycle program: 12 min preheating at 94°C, followed by for 40 regular PCR cycles, 30 sec at 94°C for denaturing, 30 sec at 59°C for annealing, and 45 sec at 72°C for elongation, and a final extension step of 7 min at 72°C. The PCR products were verified by electrophoresis in 2% agarose gel and Sanger sequencing.

### Amplification and sequencing of TTV ORF1

Entire ORF1 was amplified by using inverted long distance PCR with sense primer 5’-GTC AAG GGG CAA TTC GGG CWC-3’ and antisense primer 5’-GTC TGG CCC CAC TCA CTT TCG-3’ derived from 5’- and 3’-UTR respectively. The long distance amplicon is about 3.2 kb in length. Reaction was performed with Expand Long Template PCR System (Roche) in GeneAmp PCR System 9700 (Applied Biosystems) and PCR program was modified from previous study [12]. Shuttle PCR was designed for reducing the amount of non-full length PCR products in original program and we further coupled with touchdown cycles to reduce non-specific amplifications of long distance PCR. The whole program of long distance PCR was start with a preheating of 2 min at 94°C; followed by 5 touchdown cycles: 20 sec at 94°C for denaturing, 20 sec at 68°C for annealing which decreased by 1°C per cycle, and 2 min at 68°C for elongation; followed by 17 regular PCR cycles using the same program as touchdown cycles except the annealing step was replaced by a fixed temperature at 63°C; followed by 18 shuttle PCR cycles under the same temperature and time for denaturing and elongation of previous cycles but the annealing step was skipped; and followed by a final extension step of 5 min at 72°C. PCR products were verified in 0.7% agarose gel and purified from sliced gel band by using GFX PCR DNA and Gel Band Purification Kit (Amersham Biosciences) to avoid any non-specific amplified product. Purified PCR products were inserted into pCR-TOPO 2.1 vector (Invitrogen) and transformed into DH5α competent cells. DNA was extracted from each single colony by alkaline lysis and sequenced by primer walking approach.

### Phylogenetic analysis

To determine the phylogenetic relationship of eastern Taiwanese indigenous strains among human TTV, a total of 160 TTV ORF1 sequences from GenBank and 13 isolates from this study (S1 Appendix) were included for phylogenetic analysis. Nucleotide sequences were aligned by using the MEGA5 software on the basis of the amino acid sequences to prevent from introduction of nonsense indels [13]. The hypervariable region (HVR) and a part of 5’-end and 3’-end were excluded in subjected analysis because of the extremely high diversity in these regions resulting to an ambiguous alignment. Homologous regions of ORF1 that according to the position of prototype TA278 genomic sequence in 793–1410 nt and 1801–2595 nt were involved in subjected analysis (S1 Alignment). The best-fit evolutionary model was selected by the Bayesian information criterion using the MEGA 5 software [13]. Bayesian inference of phylogeny was carried out under the best fitting GTR+I+Γ model by MrBayes software using Metropolis coupled Markov chain Monte Carlo (MCMCMC) approach [14]. MCMCMC was performed with two parallel runs, each run with four incrementally heated (temperature = 0.1) chains for 3,000,000 generations, and sampled per 500 generations until the standard deviation of the split frequencies falls below 0.01. Bayesian tree were inferred from combined data of both runs with 10% burnin by maximum clade credibility with median node heights using TreeAnnotator v1.8.0 [15].

## Results and Discussion

TTV DNA prevalence was estimated 95% (133/140) in healthy individuals of eastern Taiwanese indigenes by detecting UTR region. Our result showed the TTV prevalence was underestimated in previous study that used N22 region for PCR detection [10]. The prevalence is similar when compared to other geographic regions via the UTR detection (Table 1). In contrast, there is a significant difference (*p* < 10^−6^) among the prevalence detected by N22 region between eastern Taiwan indigenous population and general population of southern Taiwan that is merely 150 km apart [10,16] This difference is due to the fact that PCR primers deduced from N22 region can only detect a portion of group 1 TTV of which the earliest identified strains, whereas primers derived from UTR are capable to amplify all known TTV groups. Direct sequencing of the UTR region amplicon revealed an extensive mix-infection of different TTV strains within the infected individual. The divergence of prevalence between different detecting regions suggested that the ubiquitousness of TTV is due to the co-existence of multiple phylogenetic groups. TTV is one of the most prevalent infectious agents that contributes to human virome without any disease association and some researchers have considered TTV as part of human normal flora [17]. However, it is difficult to find uninfected control to identify disease associations of TTV or their interaction of normal immune system. It is worthwhile to distinguish the specific phylogenetic group from the mix-infection pools.

**Table 1:** Comparison of the TTV’s prevalence detected by UTR region between eastern Taiwanese indigenous population and general population from other geographic regions.

Population	Total Number	Positive n (%)	Negative n (%)	p*	References
**Taiwan (Indigenes)**	140	133 (95.0)	7 (5.0)	-	This study
**Japan 1**	100	92 (92.0)	8 (8.0)	0.343857	[8]
**Japan 2**	120	112 (93.3)	8 (6.7)	0.565566	[6]
**China**	29	29 (100)	0 (0)	0.218735	[6]
**Norway**	201	180 (89.6)	21 (10.4)	0.071447	[18]
**Indonesia**	244	233 (95.0)	11 (5.0)	0.826298	[19]
**Russia**	512	485 (94.1)	27 (5.9)	0.897391	[20]

*p value of chi-square test

In order to investigate their phylogenetic relationship with other TTV strains, entire ORF1 was amplified, cloned, and sequenced from one TTV positive sample. A total of 13 isolates were obtained, and sequences were deposited in the GenBank database and assigned with accession numbers from FJ392105 to FJ392117. Our isolates can be divided into 4 data sets based on translated length and nucleotide similarity of putative ORF1 protein (both nonsense mutation and indels, that leading to reading frame shift in individual case but not present in consensus sequence, are ignored while calculating the translated length). Set 1 included 8 isolates (TW53A25, TW53A27, TW53A28, TW53A29, TW53A32, TW53A34, TW53A35, and TW53A39) and the putative ORF1 is 770 amino acids (a.a.) in length that is identical to the TTV prototype TA278. Set 2 including 3 isolates (TW53A26, TW53A30, and TW53A31) and the putative ORF1 is 736 a.a. in length. Set 3 (TW53A36) and set 4 (TW53A37) are single isolate which have a putative ORF1 with 767 and 733 a.a. in length respectively. The similarity matrix of putative ORF1 proteins that calculated by comparing between the TTV prototype TA278 and representative strain of each data set (TW53A25, TW53A26, TW53A36, and TW53A37) from our isolates was summarized in Table 2. While comparing putative ORF1, strains from the data set 1 sharing an overall nucleotide identity of 98% and the distribution of nucleotide substitutions seemed non-random. Approximately 75% of nonsynonymous mutations are restricted in the HVR, a region covering around one-sixth of ORF1’s length (Table 3). This result is similar with previous studies that HVR restricted mutational hotspots are the basic characteristics of human TTVs, regardless of phylogenetic groups [7,21].

**Table 2:** Pairwise similarity table for ORF1 amino acid sequences by comparing representative strains to TTV prototype TA278.

Representative isolate	TA278	TW53A25	TW53A37	TW53A36	TW53A26
TA278	ID	0.44	0.504	0.371	0.428
TW53A25		ID	0.441	0.393	0.704
TW53A37			ID	0.383	0.441
TW53A36				ID	0.396
TW53A26					ID

**Table 3:** Count of amino acid substitutions^a^ for putative ORF1 protein within TW53A25 sub-clade.

Isolates	TW53A27	TW53A28^b^	TW53A29	TW53A32	TW53A34^b^	TW53A35	TW53A39
**Total substitutions**	20	28	20	19	28	27	22
**HVR**^**c**^ **substitutions**	17	24	17	17	21	20	21
**HVR**^**c**^**/ORF1**	85%	86%	85%	89%	75%	74%	95%

^a^ Substitutions were compared between TW53A25 and other 7 isolates.

^b^ Putative ORF1 sequence contains a part of terminal regions that were not submitted to GenBank due to only single strain sequenced. (Sequences provided in S1 Sequences)

^c^ HVR was operationally defined as the amino acid positions between 275 and 402 of TW53A25 ORF1 protein here.

**Fig 1:**
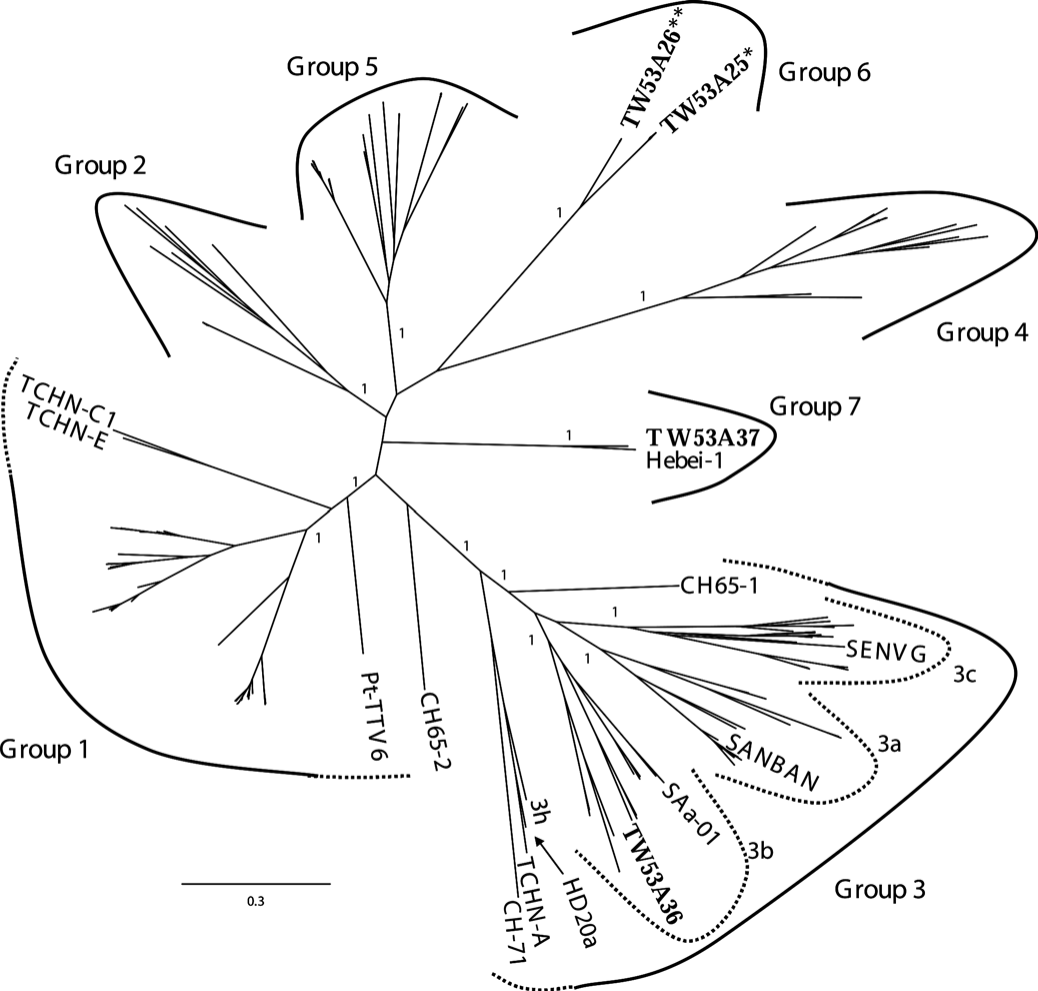
Unrooted Bayesian inferred maximum clade credibility tree of TTV ORF1 well aligned region. Posterior probabilities are labeled at major branches. Branches with a posterior probabilities equal or above 0.95 are considered as strong supported. Isolates from this study are labeled in bold. The scale bar at the left bottom indicates evolutionary distance in unit of substitutions per site per generation. The semicircular symbols indicate seven major TTV phylogenetic groups. The dash lines indicate tentatively classifications. Tentatively classified strains are labeled for reference. The SANBAN, SAa-01, and SENVG are arbitrarily chosen as representative for showing subgroup 3a, 3b, and 3c respectively. See S1 Fig and S1 Appendix for detail taxa list. *Label TW53A25 including 8 isolates (TW53A25, TW53A27, TW53A28, TW53A29, TW53A32, TW53A34, TW53A35, and TW53A39). **Label TW53A26 including 3 isolates (TW53A26, TW53A30, and TW53A31).

Bayesian phylogeny showed that TTVs were not classified into 4 but 5 groups. Five major branches were well supported by posterior probabilities ≥ 0.95 (Fig 1). Thirteen new TTV isolates from this study can be classified into 3 clades. Isolate TW53A36 was classified into group 3 whereas the other 12 isolates could not classify into any of currently known phylogenetic groups. Eight isolates of data set 1 (TW53A25 as a representative isolate) and 3 isolates of data set 2 (TW53A26 as a representative isolate) were clustered together into a discrete monophyletic clade (the “group 6”) The TW53A37 was clustered together with recently identified Hebei-1 strain (the “group 7”). Both clades were distinct related to all other previously known strains with posterior probabilities of 1.0 (Fig 1).

**Fig 2:**
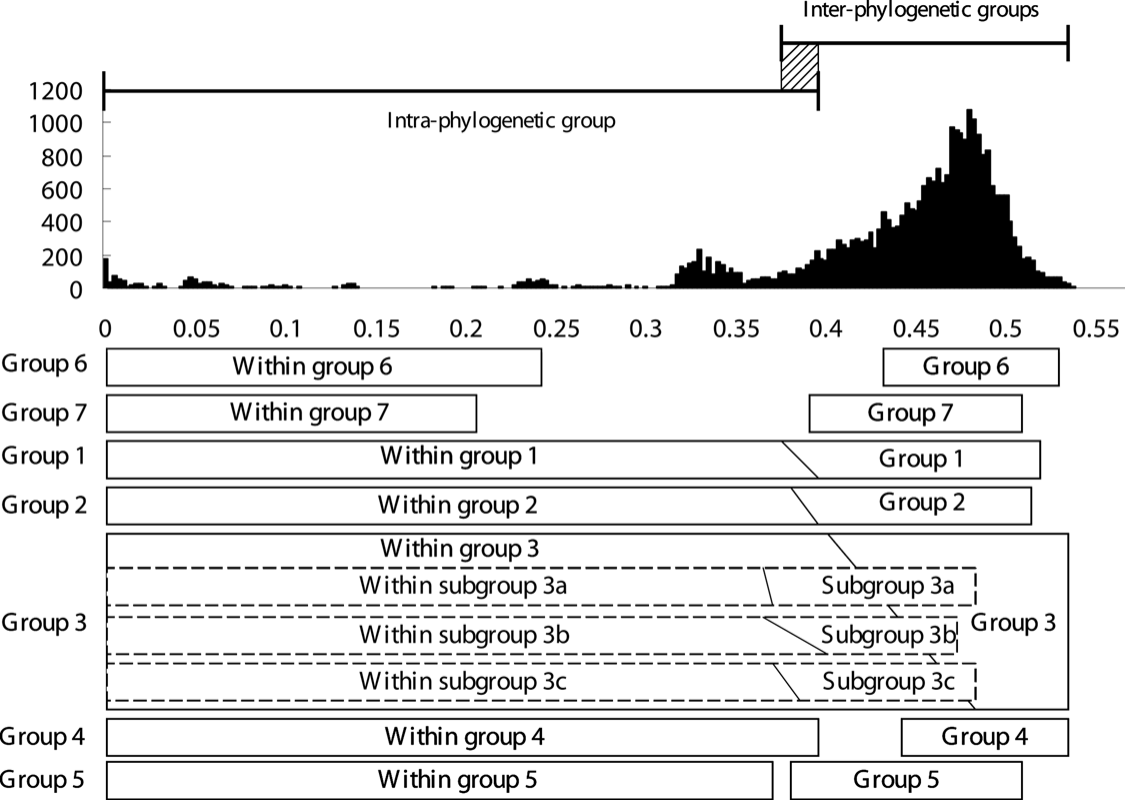
Frequency distribution plot of p-distance for TTVs. The uncorrected distance matrix is calculated from the same data set as that used for phylogenetic analysis. Y axis represents the frequency counts in each bin. X axis represents the value of uncorrected pairwise distance. Horizontal bars indicate the distribution ranges. The gray zone indicates the overlapping regions between the distribution of intragroup and intergroup distances. Rectangles indicate the distance distribution ranges of each group and divided into left and right regions. Left regions show the distribution range of which pairwise distances are calculated within each group (intragroup distances). Right regions show the distribution range of pairwise distances that compared to strains of other groups (intergroup distances). The slash lines indicate the overlapping gray area of each group. Rectangles of group 6 and group 7 have small left regions because only limited strains are available. The subgroups of group 3 (3a, 3b, and 3c) are indicated by dashed rectangles. Dashed rectangles are also divided into two parts as well as major groups, except the right regions showing the distribution range of pairwise distances that compared to strains from two other subgroups in group 3.

To place newly revealed monophyletic clades to an appropriate taxonomic level, we try to clarify the classification threshold of phylogenetic group based on the distribution pattern of pairwise distances. A matrix of uncorrected pairwise distances (p-distance) for drawing a frequency distribution plot was calculated according to previous study [5]. The distribution ranges of intra- and inter- phylogenetic groups distances were organized to represent the diversity of each group in Fig 2. In general, p-distances of 5 current known groups were distributed in less than 0.3950 and greater than 0.3750 when compared to isolates of the same group and to other groups respectively. The overlapping region from 0.3750 to 0.3950 indicated a gray area between inter- and inter-group distances, which also indicated a new strain cannot be classified into any specific phylogenetic group merely by comparing p-distance without a phylogeny. Combined the distance distributions and phylogeny of TTVs, a criterion is proposed: a set of TTV isolates should be considered as a phylogenetic group when they are clustered into a statistically supported monophyletic clade and have comparable intergroup distances to currently identified groups. Following the construction above, the distribution plots that comparing observed distance between group 6 and group 7 to all other groups was also calculated and indicated in Fig 2. Intergroup distances of both groups are ranged greater than 0.3950: group 6 shows a similar distribution to group 4; whereas group 7 shows a similar distribution to group 2. Combined this result and phylogenetic analysis, group 6 and group 7 should be considered as novel phylogenetic groups, designed as the 6th and the 7th phylogenetic group of TTV. Previous study of Jelcic et al. has reported both intra- and inter-phylogenetic group mix-infections of TTV were found in Hodgkin's disease patient [7]. Nevertheless, our data showed this phenomenon can be observed in healthy individual as well. This suggested that such mix-infection is one of the basic characteristics of human TTVs.

In addition, the group 3, the most diverse group of TTV, showed a remarkably broad gray area distributed from 0.4025 to 0.4900. When refer to phylogenetic tree, TTV group 3 can be further divided into 3 subgroups: namely 3a, 3b, and 3c. The frequency distribution plots were drawn as well as above except the p-distances were calculated by comparing isolates within each subgroup and between subgroups of TTV group 3 (Fig 2). The distribution range of subgroup 3a, 3b, and 3c present a comparable diversity to least diverted group, group 5; in contrast, when comparing each subgroup of group 3 to all other strains, there are valleys on their distribution plots which represent as a boundary between subgroups and major groups (S4 Fig). These results suggested that the group 3 should still be considered as a large group that including 3 subgroups.

**Fig 3:**
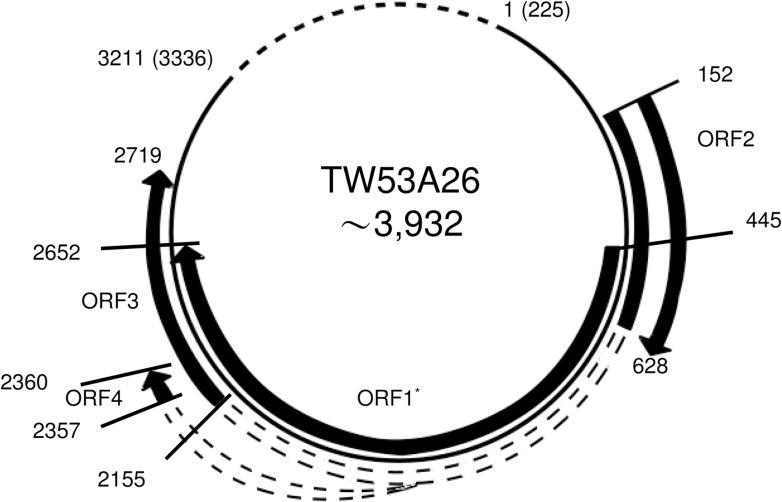
Putative genomic organization of TTV group 6 by using TW53A26 as representative isolate. ORFs are marked with black arrows. Predicted donor and acceptor sites are linked by dashed border. The dotted line indicated UTR that was not included in our ORF amplicon. ORF1 encoded for 736 amino acids (nt 445–2652), ORF2 for 159 amino acids (nt 152–628), ORF3 for 347 amino acids (nt 152–627 and 2155–2719), ORF4 for 160 amino acids (nt 152–627 and 2357–2360). The numbering of nt 1 (the first) and the nt 3211 (last nucleotide) of TW53A26 is according to nt 225 and nt 3336 of TA278, respectively. Numbers in bracket indicated the corresponding nucleotide position in TA278. The full length of TTV group 6 is about 3,932 nt calculated by assuming the UTR is conserved with Hel32, a strain with the same length of translated ORF1. *Since it is not included in consensus sequence, this group that a nonsense mutation of ORF1 at nt 523 of isolate TW53A26 is ignored here.

As the representative isolate of group 6, the putative genomic organization of TW53A26 isolate is illustrated in Fig 3. Although the amplicon of our long distance PCR did not include the template region of the first splicing site, the second (nt 627 and 2155) and the third (nt 627 and 2357) predicted donor and acceptor sites are well conserved with studied strains [22,23]. The putative ORF2 protein is 151 a.a. (nt 176–628) in length, which is about 30 a.a. longer than other TTVs. The putative ORF3 protein from TW53A25 and TW53A26 subclades are 345 and 347 a.a. in length respectively, which are slightly longer than other TTVs. The ORF4 putative protein of TW53A26 is 160 a.a. in length, which is only a half of other TTVs (about 300 aa in length). This might be due to a C→T premature mutation on nt 2361.

In conclusion, our study presents the most updated information regarding the classification of TTV and its molecular epidemiology in eastern Taiwanese indigenes. We showed extremely high prevalence of TTV infection in eastern Taiwanese indigenous population and found an extensive mix-infection manner from a healthy individual. Both intra- and inter-phylogenetic groups mix-infection can be found from a single person. In addition to the currently indentified phylogenetic groups, we found all our isolates in this study are distantly related to all other known strains based on phylogenetic analysis of the entire ORF1. A criterion based on phylogeny and the distribution pattern of p-distance was established to clarify the definition of the taxonomic level: phylogenetic group and two novel clades identified in this study are fitted to the phylogenetic group level. These new groups are designed as the 6th and 7th phylogenetic group. Our study has further broadened the field of human TTVs and proposed a robust criterion for classification of the major human TTV phylogenetic groups.

## Supporting Information

S1 Fig Unrooted Bayesian tree of TTV. It is the same tree as Fig 1 represent in rectangular form. The root placed at non-human primate strain (CH65-2) is not a real root but for easier visual of tree topology.
(EMF)

S2 Fig Distribution plot of intragroup distances. Frequency distribution plots that calculated by comparing p-distance between isolates within the same group (except group 6 and group 7).
(PPT)

S3 Fig Distribution plot of intergroup distances. Frequency distribution plots that calculated by comparing p-distance between isolates of each group to isolates of other groups.
(PPT)

S4 Fig Distribution plot of subgroup 3a, 3b, and 3c. Frequency distribution plots of intra-subgroup and inter-group distances for subgroup 3a, 3b, and 3c.
(PPT)

S1 Appendix TTV isolates and their GenBank accession Numbers. A list of TTV isolates that included in phylogenetic analysis.
(XLS)

S1 Alignment The alignment file of TTV ORF1 used in phylogenetic analysis. (FAS)

S1 Sequences The ORF1 nucleotide sequence of TW53A28 and TW53A34. (FAS)
