# Supplementary material for: New Phylogenetic Groups of Torque Teno Virus Identified in Eastern Taiwan Indigenes
Source: PLoS One. 2016 Feb 22;11(2):e0149901. doi: 10.1371/journal.pone.0149901 (PMC4762681; doi:10.1371/journal.pone.0149901)
Supplement: S2 Fig — Frequency distribution plots that calculated by comparing p-distance between isolates within the same group (except group 6 and group 7). (PPT) [file pone.0149901.s002.ppt]

## Slide 1
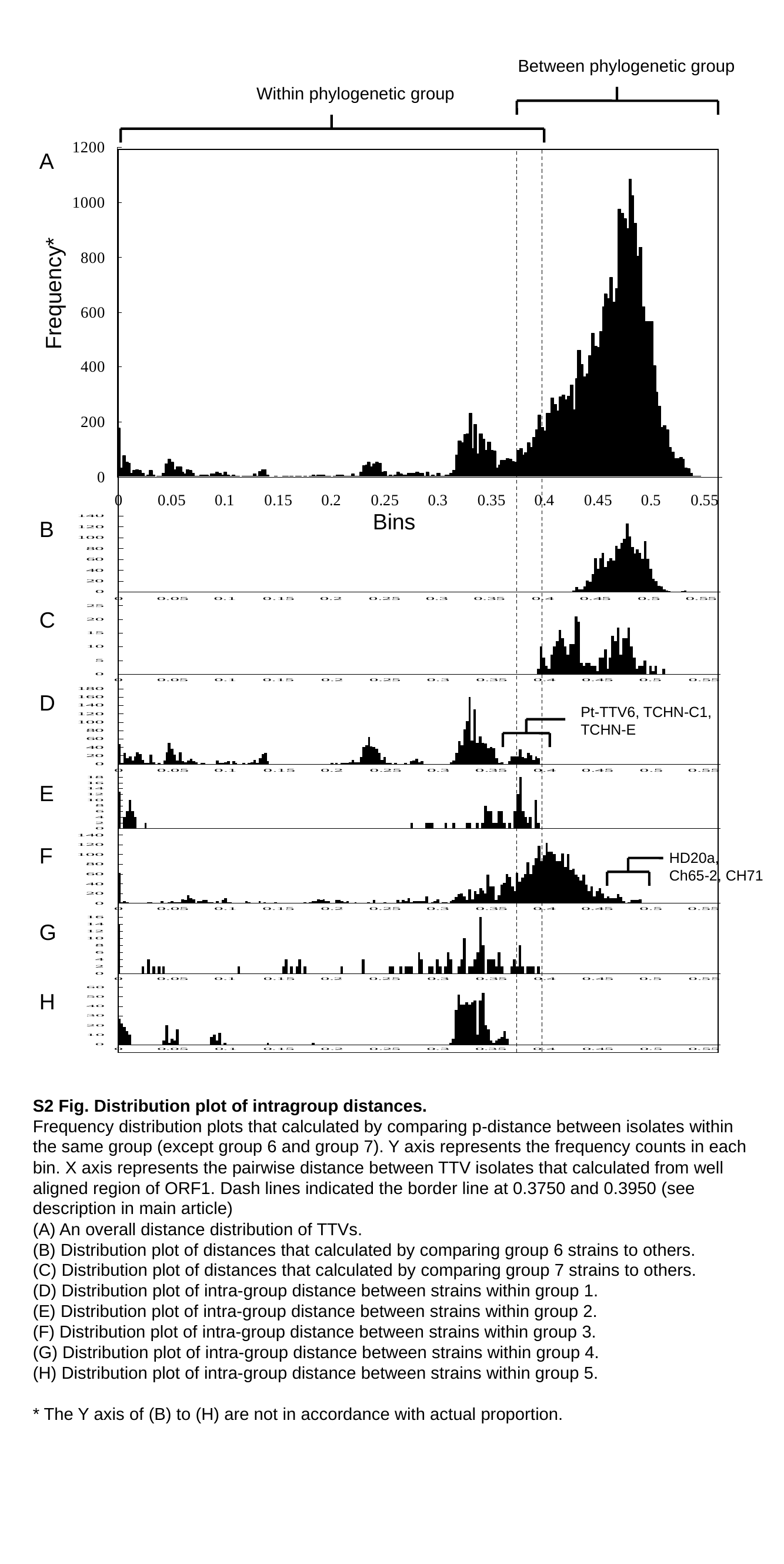

Between phylogenetic group
Within phylogenetic group
A
Frequency*
Bins
B
C
D
Pt-TTV6, TCHN-C1,
TCHN-E
E
F
HD20a,
Ch65-2, CH71
G
H
S2 Fig. Distribution plot of intragroup distances.
Frequency distribution plots that calculated by comparing p-distance between isolates within the same group (except group 6 and group 7). Y axis represents the frequency counts in each bin. X axis represents the pairwise distance between TTV isolates that calculated from well aligned region of ORF1. Dash lines indicated the border line at 0.3750 and 0.3950 (see description in main article)
(A) An overall distance distribution of TTVs.
(B) Distribution plot of distances that calculated by comparing group 6 strains to others.
(C) Distribution plot of distances that calculated by comparing group 7 strains to others.
(D) Distribution plot of intra-group distance between strains within group 1.
(E) Distribution plot of intra-group distance between strains within group 2.
(F) Distribution plot of intra-group distance between strains within group 3.
(G) Distribution plot of intra-group distance between strains within group 4.
(H) Distribution plot of intra-group distance between strains within group 5.
* The Y axis of (B) to (H) are not in accordance with actual proportion.
