# Supplementary material for: New Phylogenetic Groups of Torque Teno Virus Identified in Eastern Taiwan Indigenes
Source: PLoS One. 2016 Feb 22;11(2):e0149901. doi: 10.1371/journal.pone.0149901 (PMC4762681; doi:10.1371/journal.pone.0149901)
Supplement: S3 Fig — Frequency distribution plots that calculated by comparing p-distance between isolates of each group to isolates of other groups. (PPT) [file pone.0149901.s003.ppt]

## Slide 1
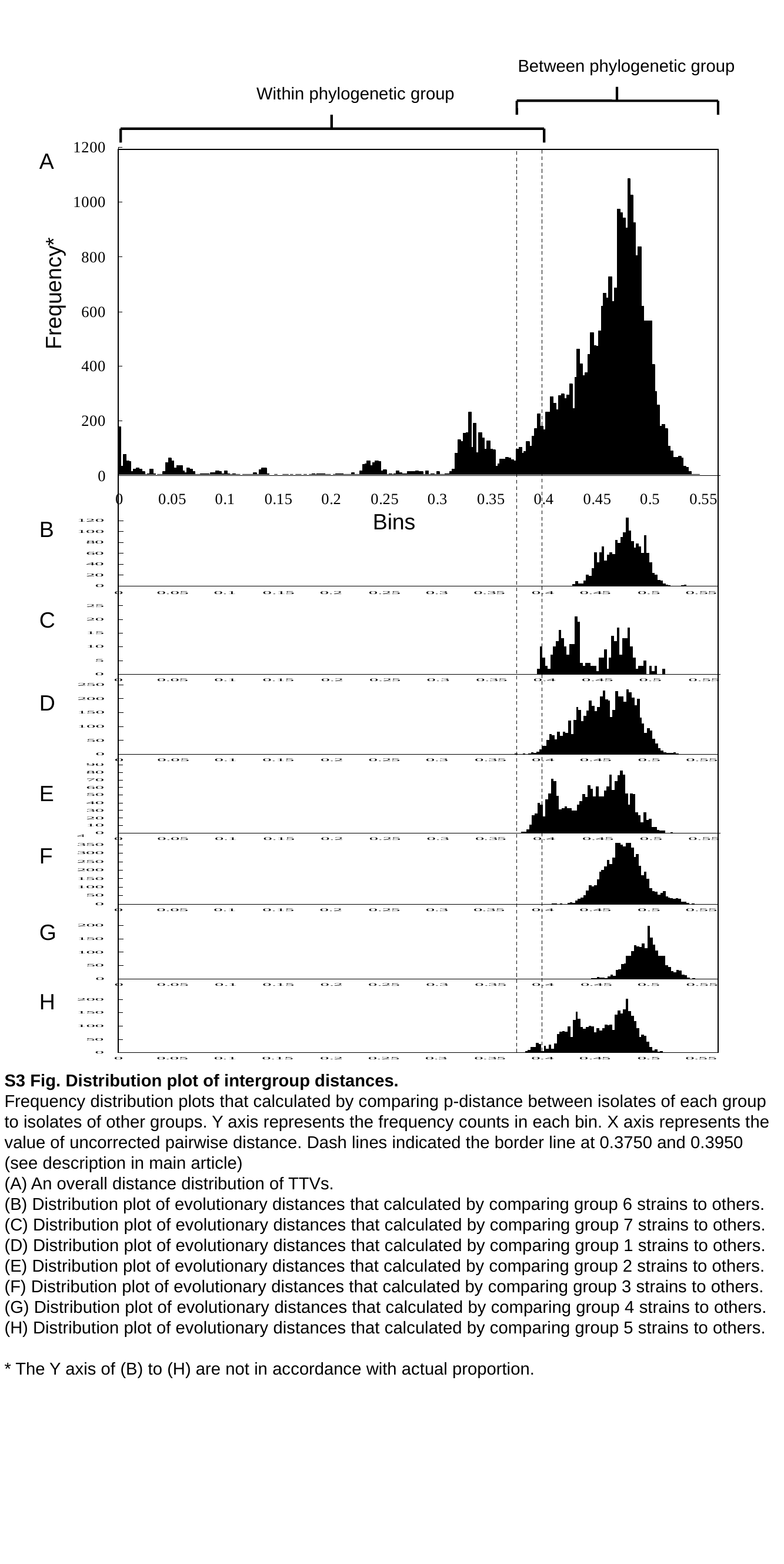

Between phylogenetic group
Within phylogenetic group
A
Frequency*
Bins
B
C
D
E
F
G
H
S3 Fig. Distribution plot of intergroup distances.
Frequency distribution plots that calculated by comparing p-distance between isolates of each group to isolates of other groups. Y axis represents the frequency counts in each bin. X axis represents the value of uncorrected pairwise distance. Dash lines indicated the border line at 0.3750 and 0.3950 (see description in main article)
(A) An overall distance distribution of TTVs.
(B) Distribution plot of evolutionary distances that calculated by comparing group 6 strains to others.
(C) Distribution plot of evolutionary distances that calculated by comparing group 7 strains to others.
(D) Distribution plot of evolutionary distances that calculated by comparing group 1 strains to others.
(E) Distribution plot of evolutionary distances that calculated by comparing group 2 strains to others.
(F) Distribution plot of evolutionary distances that calculated by comparing group 3 strains to others.
(G) Distribution plot of evolutionary distances that calculated by comparing group 4 strains to others.
(H) Distribution plot of evolutionary distances that calculated by comparing group 5 strains to others.
* The Y axis of (B) to (H) are not in accordance with actual proportion.
