# Supplementary material for: New Phylogenetic Groups of Torque Teno Virus Identified in Eastern Taiwan Indigenes
Source: PLoS One. 2016 Feb 22;11(2):e0149901. doi: 10.1371/journal.pone.0149901 (PMC4762681; doi:10.1371/journal.pone.0149901)
Supplement: S4 Fig — Frequency distribution plots of intra-subgroup and inter-group distances for subgroup 3a, 3b, and 3c. (PPT) [file pone.0149901.s004.ppt]

## Slide 1
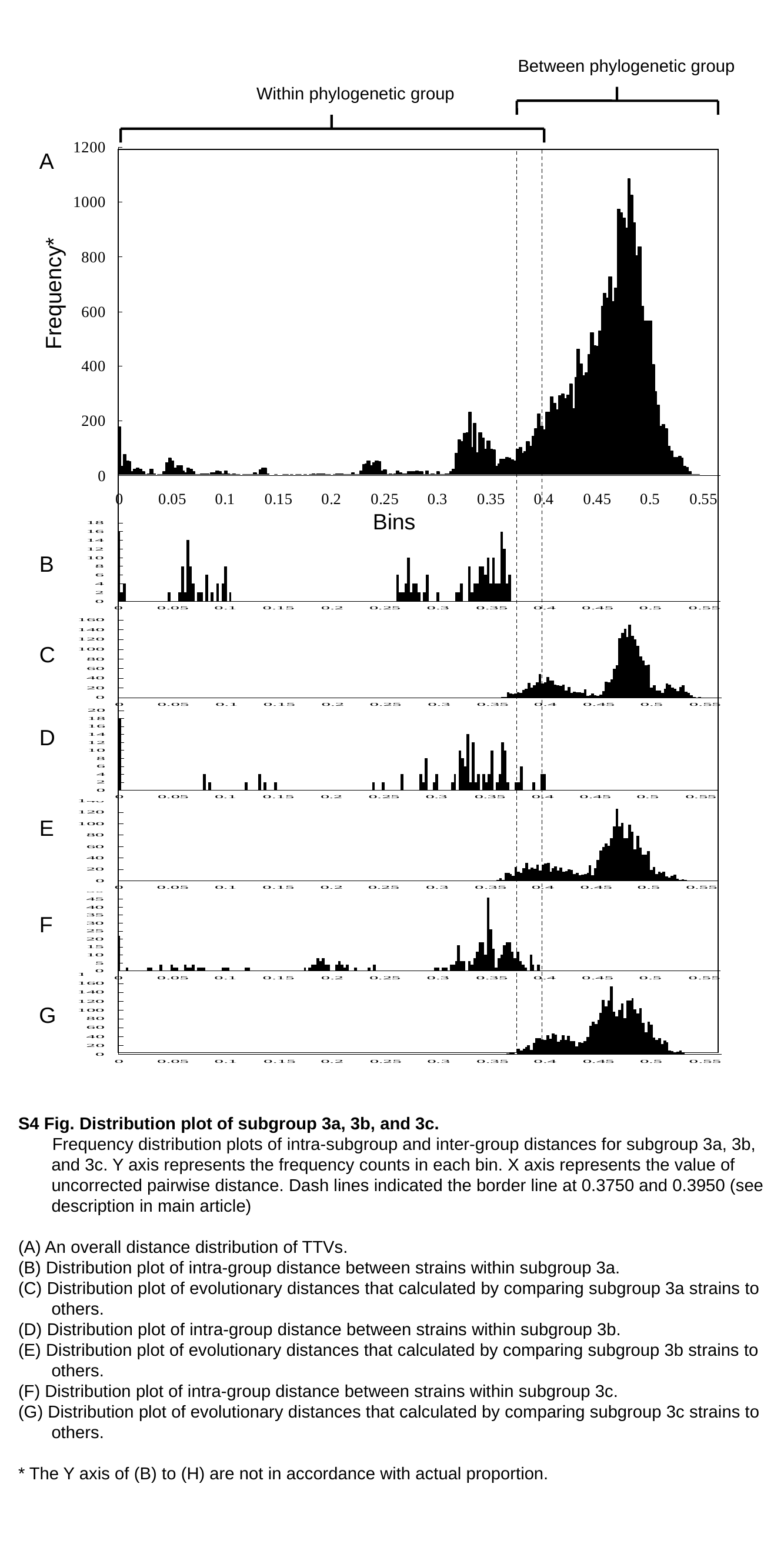

Between phylogenetic group
Within phylogenetic group
A
Frequency*
Bins
B
C
D
E
F
G
S4 Fig. Distribution plot of subgroup 3a, 3b, and 3c.
 Frequency distribution plots of intra-subgroup and inter-group distances for subgroup 3a, 3b, and 3c. Y axis represents the frequency counts in each bin. X axis represents the value of uncorrected pairwise distance. Dash lines indicated the border line at 0.3750 and 0.3950 (see description in main article)
(A) An overall distance distribution of TTVs.
(B) Distribution plot of intra-group distance between strains within subgroup 3a.
(C) Distribution plot of evolutionary distances that calculated by comparing subgroup 3a strains to others.
(D) Distribution plot of intra-group distance between strains within subgroup 3b.
(E) Distribution plot of evolutionary distances that calculated by comparing subgroup 3b strains to others.
(F) Distribution plot of intra-group distance between strains within subgroup 3c.
(G) Distribution plot of evolutionary distances that calculated by comparing subgroup 3c strains to others.
* The Y axis of (B) to (H) are not in accordance with actual proportion.
